# Efficacy and Safety of 5-Aminolevulinic Acid Hydrochloride Combined with Sodium Ferrous Citrate in Pediatric Patients with Leigh Syndrome and Central Nervous System Disorders: An Initial Exploratory Trial with a Double-Blind Placebo-Controlled Period, Followed by an Open-Label Period and a Subsequent Long-Term Administration Study

**DOI:** 10.3390/life15081168

**Published:** 2025-07-23

**Authors:** Yuichi Abe, Toshimitsu Hamasaki, Jun Natsume, Yukiko Mogami, Kei Murayama, Hideaki Shiraishi, Yuki Abe, Satoko Kumada, Ryuta Tanaka, Kenji Ihara, Takafumi Sakakibara, Yasushi Okazaki, Hitoshi Nakagawa, Kiwamu Takahashi, Mitsugu Yamauchi, Motowo Nakajima, Akira Ohtake

**Affiliations:** 1 Department of Pediatrics, Saitama Medical University Hospital, Moroyama, Saitama 350-0495, Japan; abe-yu@ncchd.go.jp; 2 Division of Neurology, National Center for Child Health and Development, Tokyo 157-8535, Japan; 3 The Biostatistics Center and Department of Biostatistics and Bioinformatics, Milken Institute School of Public Health, The George Washington University, Rockville, MD 20852, USA; thamasaki@gwu.edu; 4 Department of Pediatrics, Nagoya University Hospital, Nagoya 466-8550, Japan; junnatsu@med.nagoya-u.ac.jp; 5 Department of Developmental Disability Medicine, Nagoya University Graduate School of Medicine, Nagoya, 466-8550, Japan; 6 Department of Pediatric Neurology, Osaka Women’s and Children’s Hospital, Osaka 594-1101, Japan; yukimoga@wch.opho.jp; 7 Department of Metabolism, Chiba Children’s Hospital, Chiba 266-0007, Japan; kmuraya@mri.biglobe.ne.jp; 8 Department of Pediatrics, Juntendo University, Tokyo 113-8431, Japan; 9 Department of Diagnostics and Therapeutics of Intractable Diseases, Intractable Disease Research Center, Graduate School of Medicine, Juntendo University, Tokyo 113-8431, Japan; ya-okazaki@juntendo.ac.jp; 10 Department of Pediatrics, Hokkaido University Hospital, Sapporo 060-8648, Japan; siraisi@med.hokudai.ac.jp; 11 Department of Pediatrics, Dokkyo Medical University, Mibu, Tochigi 321-0293, Japan; 12 Department of Pediatrics, Niigata City General Hospital, Niigata 950-1197, Japan; y-abe@hosp.niigata.niigata.jp; 13 Department of Neuropediatrics, Tokyo Metropolitan Neurological Hospital, Fuchu 183-0042, Japan; satoko_kumada@tmhp.jp; 14 Department of Neurology, Ibaraki Children’s Hospital, Mito 311-4145, Japan; r-tanaka@md.tsukuba.ac.jp; 15 Ibaraki Pediatric Education and Training Station, University of Tsukuba, Tsukuba 305-8576, Japan; 16 Department of Pediatrics, Oita University Hospital, Oita 879-5593, Japan; k-ihara@oita-u.ac.jp; 17 Department of Pediatrics, Nara Medical University Hospital, Nara 634-8522, Japan; kumasan@naramed-u.ac.jp; 18 Laboratory for Comprehensive Genomic Analysis, RIKEN Center for Integrative Medical Sciences, Wako 351-0198, Japan; 19 Development Division, SBI Pharmaceuticals, Tokyo 106-6013, Japan; hinakaga@sbigroup.co.jp (H.N.); kiwtakah@sbigroup.co.jp (K.T.); myamauch@sbigroup.co.jp (M.Y.); motnakaj@sbigroup.co.jp (M.N.); 20 Department of Clinical Genomics, Saitama Medical University Hospital, Moroyama, Saitama 350-0495, Japan

**Keywords:** mitochondrial disease, Leigh syndrome, 5-aminolevulinic acid, sodium ferrous citrate, mitochondrial respiratory chain disorder

## Abstract

An explorative study was conducted to evaluate the efficacy and safety of 5-aminolevulinic acid hydrochloride combined with sodium ferrous citrate (SPP-004) in 10 pediatric patients with Leigh syndrome (LS) aged 3–24 months in 10 institutions between December 2014 and July 2019. The patients were randomized and allocated to the SPP-004 or placebo group for a 12-week double-blind period, followed by a 12-week open-label period with SPP-004 and then a long-term study of up to 180 weeks. The efficacy and safety were evaluated using the Newcastle Pediatric Mitochondrial Disease Scale (NPMDS) and adverse events (AEs), respectively. No significant differences were found between groups in NPMDS scores, but prolonged SPP-004 treatment stabilized or improved scores. During the initial double-blind phase, the serum lactate levels increased in the placebo group but not in the SPP-004 group. Over the period of prolonged treatment with SPP-004, the average serum lactate level gradually decreased to a normal level. One patient died due to heart failure, presumably due to an underlying disease. Overall, 7 out of 10 patients received SPP-004 without developing severe AEs until the termination of the long-term study. Given the severe symptoms and poor prognosis of pediatric LS, NPMDS scores were indicative of stabilization in pediatric LS patients treated with SPP-004.

## 1. Introduction

Mitochondrial diseases (MDs) are genetic conditions affecting mitochondrial function. Mitochondrial dysfunction due to genetic alterations may occur in any organ. Pediatric patients with MD have metabolic disorders caused by the dysfunction of energy production in the mitochondrial respiratory chain (MRC) [1] and often develop hyperlactatemia in the blood and cerebrospinal fluid. Pediatric MD often affects the central nervous system and other organs, such as the liver and kidneys [1]. Approximately 300 causative MD genes have been identified [2]. A recent study showed that evaluating the mitochondrial function of MD patients through the biochemical examination of MRC complex enzyme activity and oxygen consumption rate may be possible by using fibroblasts derived from a primary cell culture of a patient’s skin biopsy sample [3], although they are not yet considered to be diagnostic tools for MD.

Leigh syndrome (LS) is among the most typical MD types affecting the central nervous system, including the bilateral basal ganglia during infancy, resulting in lesions detected by brain magnetic resonance imaging (MRI) [4]. LS patients have psychomotor delays in infancy or childhood and progressively develop neurological regressions. LS is classically diagnosed by its clinical symptoms, characteristic brain MRI findings, and elevated lactate levels in the blood and/or cerebrospinal fluid; its confirmation is achieved through genetic examination, including mitochondrial DNA and nuclear DNA mutations [5]. Non-mitochondrial LS (Non-Mito LS) is known as LS due to its non-mitochondrial gene variant [6].

Despite the severity of pediatric LS, no drug has been approved to manage its symptoms worldwide [7,8]. In Japan, taurine has been approved as a therapeutic drug for stroke-like attacks of mitochondrial myopathy encephalopathy, lactic acidosis, and stroke-like episodes (MELAS); however, its safety and efficacy in children aged <13 years have not been confirmed. In the countries in the European Union, idebenone has been approved by the European Medicines Agency for Leber hereditary optic neuropathy, an MD inherited through the mother; however, this drug is only indicated for patients aged ≥12 years [7]. Pediatric LS patients are usually treated with oral multivitamins, nutrition, and supportive therapies [7,9]. Additionally, MELAS patients in Japan are treated with oral or intravenous L-arginine therapy [10,11].

Recently, 5-aminolevulinic acid (5-ALA) combined with sodium ferrous citrate (SFC) was shown to enhance heme production [12] and upregulate MRC activity in vitro and in vivo [13,14]. The bioavailability and brain penetration of orally administered 5-ALA hydrochloride (HCl) has been suggested in studies using mouse models for neurodegenerative diseases, such as ATR-X (alpha thalassemia/mental retardation syndrome X-linked) [15] and FXTAS (fragile X-associated tremor/ataxia syndrome) [16], as well as by enrolling middle-aged women with depression [17]. A supplement of 5-ALA phosphate with SFC has been marketed in Japan and several countries in East Asia and the Middle East and is known to be effective at improving sleep quality [18] and cooperativity [19]. These suggest that the oral administration of 5-ALA with SFC may be effective for MDs, including LS.

We proposed a clinical trial of 5-ALA HCl combined with SFC (SPP-004) for the treatment of pediatric LS patients with central nervous system disorders. These studies were conducted as an investigator-initiated trial to explore the efficacy and safety of SPP-004 in pediatric LS patients by comparing it to a placebo. These studies were composed of an initial 24-week exploratory study (12 weeks of blinded study and 12 weeks of open-label study [SPED-ALA-001]) and a subsequent long-term study until termination (SPED-ALA-002).

## 2. Materials and Methods

### 2.1. Study Drug and Placebo

The preclinical and Phase 1 studies on SPP-004 were performed by Covance US (Madison, WC, USA) and Covance UK (Leeds, UK), respectively (Internal Reports, SBI Pharmaceuticals, Tokyo, Japan). In the Phase 1 study, 10 British and 10 Japanese adults living in the UK were enrolled, and the maximum tolerated dose (MTD) was determined. Reproductive safety assessment using non-primates was conducted by Covance US in Madison. However, the carcinogenesis assessment with chronic administration has not yet been completed. All preclinical and Phase 1 clinical study reports are available upon reasonable request. The placebo comprised SFC and every ingredient of the study drug, except 5-ALA HCl. SPP 004 and placebo capsules were produced under GMP and provided by SBI Pharmaceuticals Co., Ltd. (Tokyo, Japan).

The Phase I clinical study can be summarized as follows. A Phase 1, randomized, double-blind, placebo-controlled, single, and multiple ascending oral dose study was conducted at two sites in the UK.

Fifty healthy Caucasian and Japanese male and female individuals aged 18–53 years were enrolled, totaling 120 participants. The participants were administered in cohorts of eight, with six of them receiving 5-ALA/SFC and two receiving a placebo. The effects of food and differing ratios of 5-ALA to SFC on single-dose pharmacokinetics were evaluated.

The adverse events (AEs) and adverse drug reactions (ADRs) reported in the multiple-dose phases of study SPP1C301 are presented in Table A1 and Table A2, respectively. In the multiple-dose phase, doses of up to 300 mg of 5-ALA and 470 mg of SFC were safe for men and reasonably well tolerated by women. Most cases of AEs were mild or moderate, with no serious events. Gastrointestinal and nervous system disorders were the most commonly reported. The Japanese participants had lower tolerance for higher single doses compared to their Caucasian counterparts, but this difference was not observed after multiple dosing.

The elevations in the liver enzyme levels, including alanine aminotransferase (ALT), aspartate aminotransferase (AST), and gamma-glutamyl transferase (GGT), were noted in five participants during the multiple-dose phase. One Caucasian participant had an elevated ALT level. The increases in the mean ALT, AST, GGT, and bilirubin levels were observed at higher doses but not at lower doses. No other clinically abnormal findings were noted in the laboratory evaluations, physical examinations, or vital signs.

### 2.2. Patients

Eligibility for the initial trial is shown in Table S1. For the initial trial, the investigators recruited eligible patients who met all the following criteria: Japanese children aged 3–24 months, clinically diagnosed as LS with characteristic symptoms or signs (bilateral and symmetrical lesion of the brain stem or basal ganglia) and progressive neurological disorders (delayed motor and mental development) as confirmed by the dysfunction of the MRC enzyme complexes or mutation of mitochondrial genes with a blood lactate–pyruvate ratio of ≥15.0, and with written informed consent from the surrogate (parental guardian or guardian). Meanwhile, patients who met any of the following criteria were excluded: those with cardiomyopathy, severe cardiac dysfunction, renal dysfunction, sepsis, drug allergy, or hypersensitivity to a study drug component; those who participated in another trial within 12 weeks before obtaining informed consent; and/or those who were deemed to be ineligible for study participation by the investigators. In the long-term study, patients who completed the initial trial were enrolled after obtaining informed consent.

### 2.3. LS Causative Gene Analysis

Skin fibroblasts or myoblasts were prepared by culturing the patients’ biopsy tissues and were subjected to nuclear and mitochondrial gene analyses using polymerase chain reaction primers for each known mutation in the LS causative genes according to the established protocol [20].

### 2.4. Study Design

**Figure 1:**
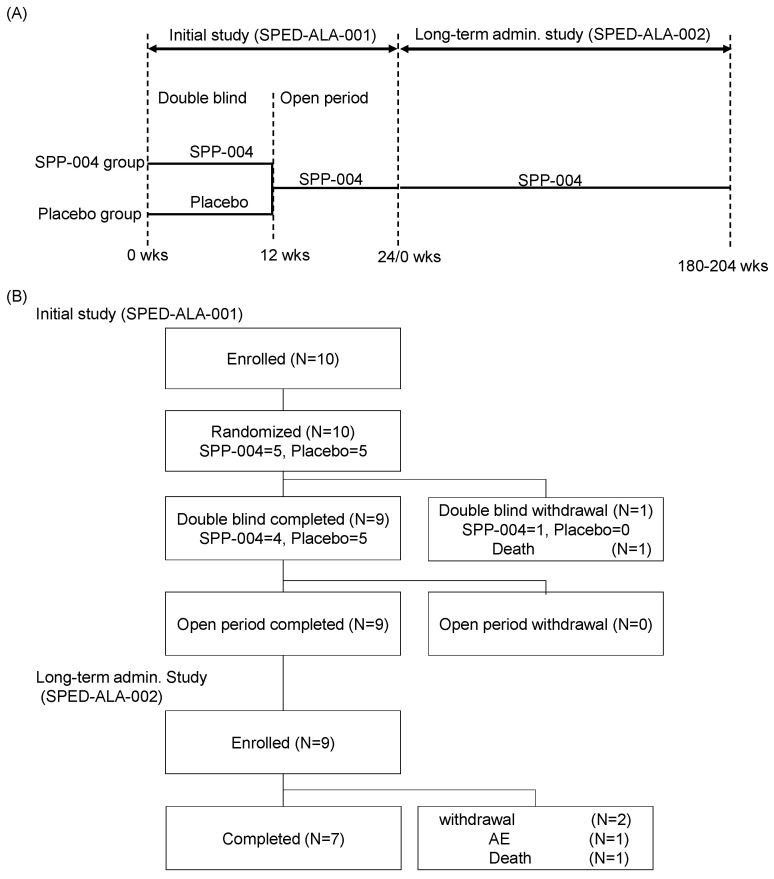
(**A**) Study design. (**B**) Flow of subject disposition.

The initial study was an exploratory clinical trial conducted as a multicenter, double-blind, and randomized test with an initial 12-week placebo-controlled period, followed by a second 12-week open-label period (SPED-ALA-001). The first double-blind period was set up to avoid leaving the placebo group untreated, considering the severe symptoms of pediatric LS. The administration of the study drug was continued in the subsequent long-term study up to termination (SPED-ALA-002) (Figure 1A). The long-term study was conducted as a multicenter, unblinded, and non-randomized study.

All trials were conducted in 10 institutions in Japan. The patients were randomly assigned to the SPP-004 or placebo group in a 1:1 ratio and given the masked study drug or placebo during the first 12 weeks. In the next 12 weeks, all participants received SPP-004 (Figure 1A), which was continued for 180–204 weeks depending on the duration of enrolment.

Considering the rarity and severity of LS and the trial’s feasibility, the target sample size was set to 10 patients because only 27 patients under 24 months old were living and eligible to participate in the initial trial as of March 2014.

The number of patients might be insufficient to detect statistical differences in the efficacy between the two groups, as the effects of SPP-004 and placebo on the total score of Newcastle Pediatric Mitochondrial Disease Scale (NPMDS) sections I–III were unknown at the time of planning. Therefore, the initial study aimed to at least determine a trend in the efficacy of SPP-004 in comparison to the placebo.

The allocation ratio was set to 1:1 to maximize the power of the test. In the initial study, all people involved (patients, investigators, and other study personnel) were blinded, except for the allocation manager for the study drug. The aim of the unblinded, single-arm, and long-term study was to determine the safety and efficacy of long-term SPP-004 administration in pediatric LS patients.

These studies were registered at https://jrct.mhlw.go.jp/ [Trial IDs jRCT2091220200 (SPED-ALA-001) and jRCT2091220214 (SPED-ALA-002)].

### 2.5. Drug Administration Protocol

In the initial study, SPP-004 or a placebo was administered orally or via a feeding tube twice a day. To be indistinguishable during the double-blind period, both drugs were masked by capsules. The SPP-004 capsule contained 25 mg 5-ALA HCl, whereas the SFC capsule contained 39.22 mg SFC. The SPP-004 group was administered 5-ALA and SFC capsules, whereas the placebo group received an SFC and a placebo capsule without 5-ALA HCl. In the long-term study, all patients received oral SPP-004 with the same doses, which were comparable to those in the initial study based on body weight (Table A3).

The doses were determined based on the results from the Phase 1 study involving Caucasian British and Japanese adults. Thanks a lot. All authors including Akira Ohtake confirmed your change.

### 2.6. Dose Setting

From a toxicological point of view, we decided to decrease the MTD dose utilized in the Phase 1 study by one level, resulting in 150 mg of 5-ALA HCl twice a day for an adult. Assuming an average adult body weight of 60 kg, this corresponds to a daily dose of 5 mg/kg body weight. Therefore, the safe doses of 5-ALA HCl administered twice a day for children aged 3 months (body weight: 6 kg) and 2 years (body weight: 12 kg) were 28.5 mg (4.75 mg/kg) and 42 mg (3.5 mg/kg), respectively.

A single dose for a 2-year-old child (body weight: 12 kg) was calculated based on the results from various pharmacological studies. For example, a previous study on the efficacy effects of 5-ALA HCl on skin fibroblasts harvested from children with MD suggested that approximately 11.7 mg of 5-ALA HCl would be suitable for a single dose [13]. Conversely, the effective dose for a 2-year-old child with a weight of 12 kg was approximately 54 mg of 5-ALA HCl, as determined in a rat complex I deficiency model induced by the injection of rotenone in the substantia nigra (SBI Pharmaceuticals Internal Reports; unpublished).

A single dose of 25 mg 5-ALA HCl/body was determined because it did not exceed the safe dose based on the toxicology study and was equal to the effective dose calculated from the pharmacology study results. Therefore, a single SFC dose was set to 39.22 mg, corresponding to a 0.5-fold molar amount of 25 mg 5-ALA HCI based on the molar ratio of 5-ALA HCl to SFC (1:0.5) in the Phase 1 study involving adults.

Given that the participants in the initial study were aged 3 months–2 years with a body weight of 6–12 kg, the 5-ALA HCl doses per body weight were 4.2–8.3 mg/kg/day. As mentioned above, the safe doses were 28.5 mg (4.75 mg/kg) and 42 mg (3.5 mg/kg) for children aged 3 months and 2 years, respectively, and the daily doses were 9.5 and 7 mg/kg, respectively.

### 2.7. Pharmacokinetic Analysis of the Significance of the Simultaneous Administration of 5-ALA and SFC

Points (1) to (3) below suggest that the simultaneous administration of 5-ALA and SFC is necessary for treating MD. (1) The amount of heme in the organ culture increased with 5-ALA alone and further increased in combination with iron [21]. This coincided with the observation that the expression of heme oxygenase-1 triggered by heme production was upregulated by the addition of 5-ALA and SFC in the cultured skin fibroblasts harvested from the biopsy samples of MD patients [13]. (2) The maximum respiration rate and ATP production in the skin fibroblasts derived from MD patients increased with the addition of 5-ALA alone; these were further increased with the simultaneous addition of 5-ALA and SFC [13]. (3) Protoporphyrin IX (PPIX) accumulation on mouse skin after the topical administration of 5-ALA HCl-containing ointment was decreased by the concomitant administration of ferrous citrate, suggesting that this type of administration could help avoid photosensitivity [22]. Furthermore, the addition of SFC suppresses PPIX accumulation in the normal cells treated with 5-ALA HCl due to the higher mitoferrin expression in the normal cells [23].

### 2.8. Pharmacokinetic Analysis

Regarding the pharmacokinetics of 5-ALA and PPIX, the blood samples were collected from patients in week 13 (pre-dose and at 0.5, 1, 2, 4, 6, and 8 h after the administration of SPP-004) and study week 24. The plasma concentrations of 5-ALA and PPIX were measured by high-performance liquid chromatography, as described by Ota et al. [24], and the pharmacokinetic parameters (Cmax, tmax, t_1/2_, AUC _0-8_, AUC_0-t_, and AUC_0-∞_) of each patient and their summary statistics (mean, standard deviation [SD], median, minimum, and maximum) were calculated.

### 2.9. Efficacy and Safety

The efficacy of SPP-004 was mainly evaluated based on the evaluation items of NPMDS [25]. The age-specific NPMDS (for 0–24 months and 2–11 years) was carefully translated to Japanese so that all the evaluators could similarly evaluate the NPMDS items. All the participants were scored and evaluated using the NPMDS at the beginning and at 8 and 12 weeks of the double-blind period and at 20 and 24 weeks of the open-label period. During the long-term study, patients were evaluated and scored using NPMDS at the beginning and every 12 weeks thereafter until termination.

The primary endpoint of these studies was the total score of NPMDS sections I–III at the time of planning. However, NPMDS section II and section III items 1 and 2 were not suitable for the evaluation of the results of these studies, as their substantial observation periods (3, 4, and 6 months) were longer than that of the study period (12 weeks for both the double- blind and open-label periods). Therefore, in addition to the original primary endpoint, the total score for section I and section III items 3–8 was also evaluated and subjected to post hoc analysis. Section I and section III items 3–8 are as follows: hearing, communication, and movement (section I), as well as vision, ptosis and eye movement, myopathy, pyramidal, and extrapyramidal, and neuropathy (section III). Self-care and ataxia were not included in the total score because they were not evaluated in patients aged <2 years.

As secondary endpoints, the scores of NPMDS sections I–IV, prognosis, blood fibroblast growth factor 21 (FGF21) level, body weight, height, and head circumference were monitored. The blood FGF21 levels were measured by using ELISA kits (BioVendor, Brno, Czech Republic). The mitochondrial respiratory chain enzyme activity of the skin fibroblasts derived from patients’ skin was also evaluated [13]. Serum lactate and pyruvate levels were measured by standard methods using equipment in the hospital’s central clinical laboratory at each institution. The acquisition and analysis of MRI images were performed according to the protocols by Saneto et al. [4] and Baertling et al. [26].

Regarding safety, AEs were defined as clinically unfavorable or unintended signs (including clinically significant laboratory abnormalities), symptoms, or diseases observed after the administration of the study drug. ADRs were defined as AEs with “a possible causal relationship with the study drug.” Serious AEs (SAEs) were defined as death, a life-threatening event, hospitalization (or prolonged stay) for treatment, a disability, a threatening disability, and a congenital anomaly in offspring, among others. Aggravations of underlying diseases other than SAEs were not evaluated as AEs. The AEs were followed up until the patient recovered or their condition returned to their pre-onset status. However, if valid, the investigator terminated the follow-up after recording the reason for termination.

### 2.10. Statistical Analysis

All efficacy endpoints were analyzed based on the full analysis set (FAS), which was as complete and as close as possible to the intention-to-treat ideal of including all randomized participants. The primary objective of the initial study was to determine whether SPP-004 is feasible and safe and to provide preliminary estimates of the treatment effect in pediatric LS patients. All endpoints were summarized using appropriate descriptive statistics. For the primary endpoint, we calculated the mean in each group, the mean differences between the two groups, and their 95% confidence intervals (CIs) for the change in the total score of NPMDS section I and section III items 3–8 from that at baseline. The Wilcoxon rank sum test was used to compare the change in the total score between the two groups.

The AEs were coded using MedDRA/J Ver. 19.0 (initial study) and Ver. 22.1 (long-term study). The clinical laboratory values (blood chemistry, hematology, and urinalysis) were determined during the observation period at 1-, 12-, 13-, and 24-week post-dose in the initial study and every 12 weeks thereafter up to the discontinuation of the drug in the long-term study. The AEs were evaluated from the start of the study drug’s administration at 24 weeks in the initial study and further monitored until the time of termination of the long-term study. For each AE, the investigators recorded the event name, onset date, severity, causal relationship to the study drug, outcome, and outcome date in the case report form. The frequency and incidence of AEs, SAEs, and ADRs were calculated for the total cohort and each group in the safety population (SP), including all randomized patients receiving at least one dose of the study drug. For the continuous values, the summary statistics were obtained from the total number of patients, each group, and each time point. For the categorical values, frequencies, and ratios, the data in each group and each evaluation time point were counted.

All reported *p*-values were two-sided, and *p* < 0.05 based on a two-sided test was considered significant. All analyses were performed using SAS software version 9.4 for Windows (SAS Institute, Inc., Cary, NC, USA).

### 2.11. Clinical Trial Institution

The clinical trials were conducted at the following hospitals: Department of Pediatrics, Saitama Medical University Hospital; Department of Pediatrics, Nagoya University Hospital; Department of Pediatric Neurology, Osaka Women’s and Children’s Hospital; Department of Metabolism, Chiba Children’s Hospital; Department of Pediatrics, Hokkaido University Hospital; Department of Pediatrics, Niigata City General Hospital; Department of Neuropediatrics, Tokyo Metropolitan Neurological Hospital; Department of Neurology, Ibaraki Children’s Hospital; Department of Pediatrics, Oita University Hospital; and Department of Pediatrics, Nara Medical University Hospital.

## 3. Results

### 3.1. Patient Enrolment and Pharmacokinetics of 5-ALA

Altogether, 10 pediatric LS patients with central nervous system disorders who were admitted at 10 institutions between 10 December 2014 and 15 September 2015 and who met all inclusion criteria were enrolled and randomly assigned to the SPP-004 (*n* = 5) and placebo (*n* = 5) groups. All patients received their assigned study drug during the double-blind period, with one patient in the SPP 004 group being excluded due to death and the remaining patients having completed the entire treatment period by March 6, 2016 (Figure 1B). All 10 patients were included in the FAS, PPS, and SP groups. The demographic characteristics of the patients (FAS) in the SPP-004 and placebo groups are shown in Table 1. The median ages of the patients in the SPP-004 and placebo groups were 20.0 (range: 15–23) and 19.5 (range: 16–23) months, respectively.

All patients were diagnosed with LS; their genetic backgrounds are shown in Table 2. Except for PLA-06-01, whose causative gene mutation was not identified, the deficiencies found in the pediatric LS patients included the following genes: *MT-ND3*, *MT ND5*, *MT-ND6*, *TRMT5*, *MT-ATP6*, *ECHS1*, *NDUFV2*, *SCN8A*, and *SURF1*. Among them, ALA-09-01 with an *SCN8A* mutation was diagnosed with Non-Mito LS (Table 2). The SPP-004 group had higher baseline NPMDS than the placebo group, suggesting that it included more severely symptomatic patients (Table 2).

**Figure 2:**
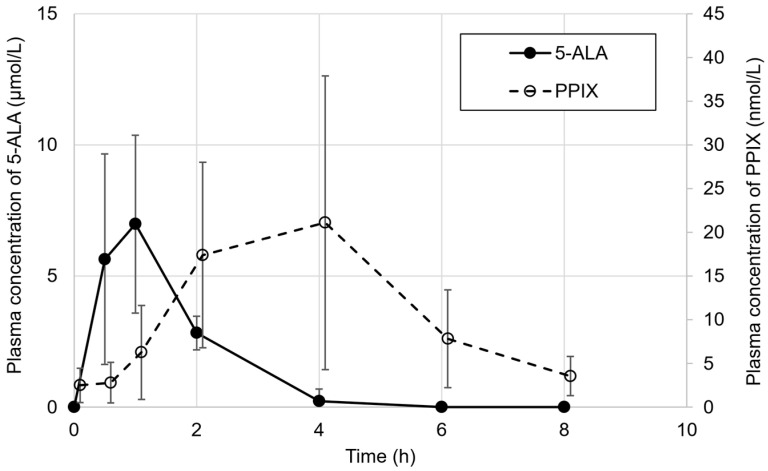
Pharmacokinetics of 5-ALA and PPIX in patients.

The pharmacokinetics of oral 5-ALA were monitored in all patients. As shown in Figure 2, the plasma 5-ALA level reached the maximum concentration (mean = 7.00 µmol/L; Table 3) at 1 h and completely disappeared at 4 h. The plasma PPIX concentration was also monitored by measuring its fluorescence (excitation light wavelength: 405 nm; emission light wavelength: 630 nm) (Figure 2). The plasma PPIX concentration gradually increased over 4 h after the oral administration of 5-ALA and nearly disappeared from the plasma after 8 h. The plasma PPIX concentration varied among the patients, with the maximum concentrations ranging from 6.58 to 49.2 nmol/L (Table 3).

### 3.2. Safety Results

During the initial study, 29 AEs occurred in five patients in the SPP-004 group, with 17 and 12 events occurring in the double-blind and open-label periods, respectively (Table 4). Meanwhile, 45 AEs occurred in five patients in the placebo group, with 21 events occurring in five patients during the double-blind period and 24 events occurring in five patients during the open-label period (Table 4).

Four ADRs (severe left ventricular failure, pulmonary alveolar hemorrhage, respiratory failure, and mild diarrhea) occurred in two patients in the SPP-004 group during the double-blind period (Table 5). In the open-label period, two ADRs (mild electroencephalogram abnormality and hematochezia, with one case each) occurred in two patients in the SPP-004 group. In the placebo group, three patients developed ADRs: one case of mild diarrhea in the double-blind period as well as two cases of moderate urticaria and one case of mild tooth discoloration in the open-label period.

There were 12 SAEs in the SPP-004 group, with seven events occurring in two patients in the double-blind period and five events occurring in two patients in the open-label period (Table S2).

Among the SAEs, left ventricular failure, pulmonary alveolar hemorrhage, respiratory failure, and hyperglycemia, which led to death, occurred in one patient in the SPP-004 group (ALA-02-02) at the early stages of the double-blind period.

The relationship to the study drug could not be excluded from these SAEs, except for hyperglycemia. No autopsy was performed, but the exacerbation of the underlying disease was strongly suspected.

In addition to the SAEs that led to fatality, two events, i.e., bronchitis and asthma, were noted in one patient, along with one event in one patient each with pharyngitis, pneumonia, gastroenteritis, and hypoxia as SAEs. In the placebo group, eight SAEs were noted, with three SAEs occurring in three patients in the double-blind period and five events occurring in one patient in the open-label period. Those symptoms were two events in one patient of epilepsy and one event in one patient each with bronchitis, generalized tonic–clonic seizure, gastroenteritis rotavirus, nasopharyngitis, pneumonia, and urticaria.

During the long-term study, the AEs and SAEs were further monitored (Table 4 and Table 5). Overall, nine patients developed 255 AEs, with seven patients developing 61 SAEs. However, the AEs related to the investigational drugs were observed in only two patients. One patient discontinued the SPP-004 treatment due to SAEs after 12 weeks; she was diagnosed with urticaria. One patient with severe symptoms died due to the causative disease after 132 weeks (Table 4). Two patients had mild skin and subcutaneous tissue disorders and urticaria, which may be related to the investigational drugs (Table 5). Tables S2 and S3 present the details of the SAEs during the initial and long-term studies.

### 3.3. Efficacy Results

**Figure 3:**
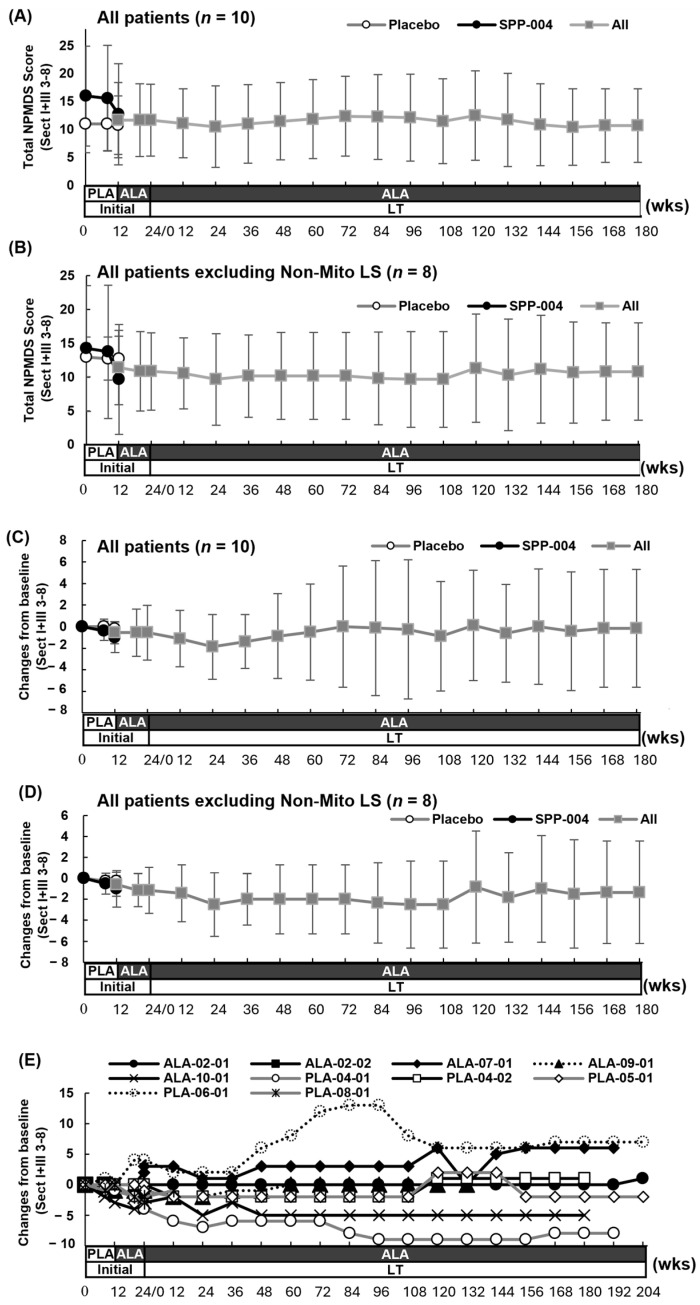
Changes in total NPMDS scores (section I and section III items 3-8). (**A**,**B**) Mean ± SD of each dose group. (**A**) All patients (*n* = 10). (**B**) All patients, excluding Non-Mito LS patients (*n* = 8). (**C**,**D**) Mean ± SD of change from baseline of each dose group. (**C**) All patients (*n* = 10). (**D**) All patients, excluding Non-Mito LS patients (*n* = 8). (**E**) Change from baseline in each patient. Dotted lines show the change in Non-Mito LS patients. ALA: SPP-004, PLA: Placebo, P2: SPED-ALA-001 study, and LT: SPED-ALA-002 study. The total score includes hearing, communication, and movement (section I), as well as vision, ptosis and eye movement, myopathy, pyramidal, extrapyramidal, and neuropathy (section III).

Figure 3 and Table S4 show the changes in the total scores of NPMDS section I and section III items 3–8 in the initial and long-term studies. Figure 3A,C show the total scores of 10 LS and Non-Mito LS patients, and Figure 3B,D show those of eight patients with LS only. During the double-blind period, the total score for the SPP-004 group tended to decrease (16.0 ± 8.94 [*n* = 5] at baseline, 12.8 ± 9.07 [*n* = 4] at week 12), whereas that of the placebo group was unchanged (11.0 ± 5.15 [*n* = 5] at baseline, 10.8 ± 5.22 [*n* = 5] at week 12) (FAS). There was no significant difference between the baseline values and scores at each time point for both groups (*p* > 0.05, Wilcoxon sum rank test).

From the open-label period of the initial study to the long-term study, all patients were continuously administered SPP-004, and the total NPMDS score was maintained despite the fluctuations in scores depending on the time points (Figure 3E). The temporary changes in the total score in some patients may be the cause of such fluctuations in the average score.

The total scores varied from 3 to 25 at the beginning of the double-blind period (Figure 3A,B). The SPP-004 group showed higher scores (mean [range]; SPP-004:16 [7,8,9,10,11,12,13,14,15,16,17,18,19,20,21,22,23,24,25] vs. placebo:11 [3,4,5,6,7,8,9,10,11,12,13,14,15,16]), and the patient (ALA-02-02) with the highest score of 25 died after the initial 8 weeks without any score changes.

During the double-blind period, the scores decreased in two patients in the SPP-004 group (i.e., symptom improvement: −2 and −3, respectively), and three patients maintained their scores without any changes for 12 weeks (Figure 3E). In the placebo group, only one patient had a lower score (−1), whereas the other four patients had unchanged total scores during 12 weeks of the double-blind period (Figure 3E). After switching from the placebo to SPP-004, the scores decreased in two patients (i.e., symptom improvement: −2 and −3, respectively), and the score increased in one patient (i.e., worsening of symptom: +4) (Figure 3E).

At each evaluation point, there were no significant differences in the changes in the total score of the NPMDS section I and section III items 3–8 between the two groups (*p* > 0.05 at 12 and 24 weeks, Wilcoxon rank sum test; Figure 3C,D). However, the total scores tended to improve in the SPP-004 group, except in one patient whose score worsened at 24 weeks in the double-blind period (Figure 3E).

When the patients were classified by the disease onset time, patients whose onset was earlier than 6 months of age had generally higher total scores (mean [range]: <6 months, 18.6 [11,12,13,14,15,16,17,18,19,20,21,22,23,24,25] vs. >6 months, 8.4 [3,4,5,6,7,8,9,10,11,12,13,14,15,16]), suggesting severe disease conditions (Figure S1A). Contrarily, the decreasing trend in the total scores, representing a response to SPP-004, appeared to be greater in the late-onset group than in the early-onset patients (mean of score change [min, max]: >6 months, −2 [−3, 0] vs. <6 months, 0.6 [−4, +4]) (Figure S1B).

The change in the total score of the NPMDS section I and section III items 3–8 was also examined among patients stratified by the class of LS causative gene mutations that were determined by nuclear and mitochondrial DNA analyses of the patients’ skin fibroblasts or myoblasts. The decrease in the total score (i.e., improvement) was similar for patients with mitochondrial DNA mutations in *MT-ND3*, *MT-ND5*, *MT-ND6*, *TR-MT5*, or *MTATP6* and those with nuclear DNA mutations in *ECHS-1*, *NDUFV2*, *SCN8A*, or *SURF1* after the SPP-004 treatment (Figure S2).

**Figure 4:**
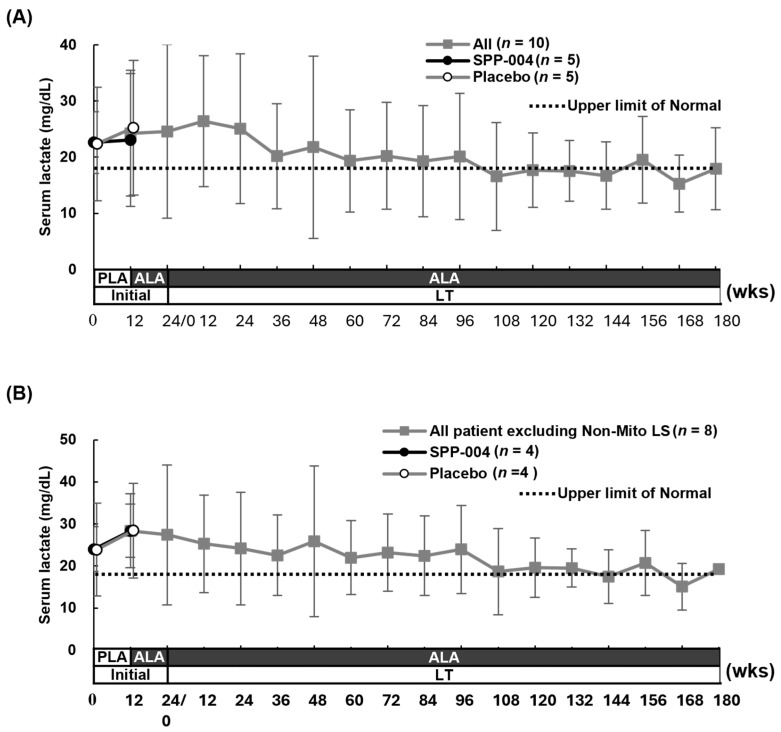
Changes in serum lactate. (**A**) All patients. (**B**) All patients, excluding Non-Mito LS patients. The average ± SD is shown. The dotted line indicates the upper limit of the normal range (18.0 mg/dL).

The serum lactate levels are shown in Figure 4 (4A, all enrolled patients, *n* = 10; 4B, LS patients, *n* = 8). During the initial double-blind period, the placebo group showed increases in serum lactate levels, whereas the SPP-004 group showed a gradual decrease. After the long-term treatment with SPP-004, the serum lactate levels normalized and stabilized up to 180 weeks.

## 4. Discussion

This 24-week exploratory initial trial (SPED-ALA-001), followed by a prolonged treatment period of up to 204 weeks (SPED-ALA-002), demonstrates the preliminary safety of 5-ALA HCl and SFC in pediatric LS patients. Additionally, certain levels of efficacy were suggested by using the total score of NPMDS section I and section III items 3–8 as the primary endpoint, although 2 out of 10 patients died due to the causative disease.

One patient in the SPP-004 group died during the early stages of the initial study. Although a causal relationship to SPP-004 could not be excluded, it was strongly suggested that the death was due to heart failure caused by the underlying disease, considering that this case was the most severe with an extremely high blood FGF21 level (Figure S3). All the other SAEs and ADRs during the initial study were confirmed to have improved or been resolved. Most AEs were associated with the primary diseases, and no AE was biased toward the SPP-004 group. Despite the limited number of cases, the twice-a-day administration of 5-ALA HCl and SFC for 24 weeks did not differ from the placebo in terms of safety in LS pediatric patients.

During the long-term study (SPED-ALA-002), one patient withdrew from this study due to urticaria. Another patient died due to respiratory problems after daily treatments with SPP-004 for 132 weeks; however, the relationship between death and treatment with SPP-004 remains unknown for this case. Abnormal increases in blood FGF21 levels were not observed before the patient died, unlike those observed in the first patient who died, suggesting that the patient’s death was caused by unexpected respiratory problems.

Lim et al. [27] reported that British children in the cohort gained an annual average of 4.5 points for the NPMDS (SD = 6.5, 95% CI = 3.0–6.1). In our study, the total score of NPMDS section I and section III items 3–8 decreased or remained unchanged in the SPP-004 group after 24 weeks. There were changes in the scores of NPMDS section I and section III items 3–8 over the long-term study period; however, no acute changes in the scores were observed, except in one patient who had substantially decreased NPMDS scores until 36 weeks in the long-term study, which markedly increased for the next 60 weeks before returning to the baseline scores. Although there might be a genetic difference between the British and Japanese children, the efficacy of SPP-004 in stabilizing and/or improving the LS symptoms may be deduced from our observations.

Ogawa et al. [28] reported that patients with (before 6 months of age) LS had a significantly higher mortality rate than those with late-onset (after 6 months of age) LS (*p* < 0.0001), suggesting the presence of severe symptoms. Lim et al. [27] also demonstrated that the probability of severe disease burden (NPMDS > 25 points) was higher in patients with early disease onset than in those with late disease onset (χ^2^ = 13.5, *p* < 0.001). Consistent with these observations, in our trial, the average total score of NPMDS section I and section III items 3–8 was higher in patients with early disease onset (i.e., severe symptoms) than in those with late disease onset. Additionally, consistent with these observations, the fatality in the SPP-004 group (ALA-02-02) had one of the earliest onsets (1 month of age) and had the highest total score of NPMDS section I and section III items 3–8 (25, i.e., most severe) at baseline. Therefore, an early disease onset may be associated with poor prognosis and severe symptoms.

**Figure 5:**
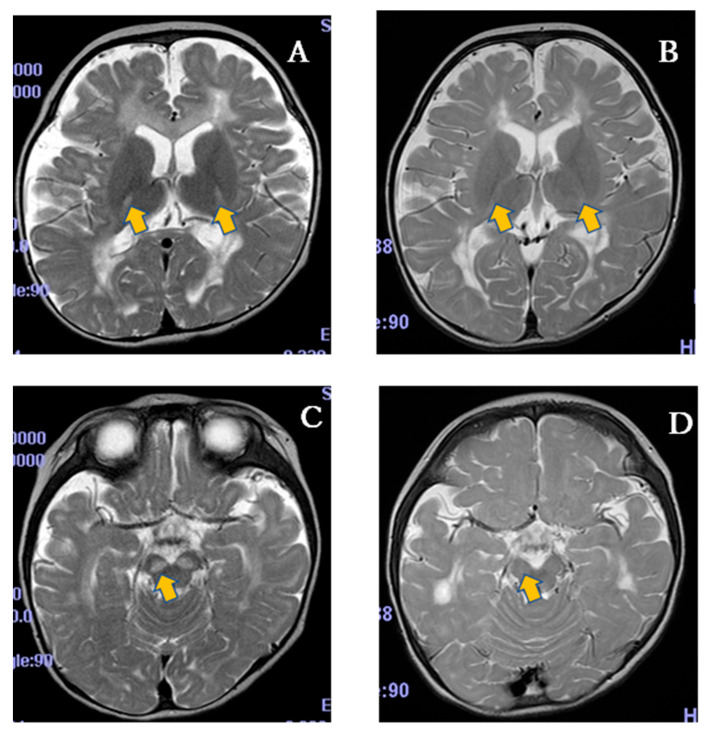
Magnetic resonance imaging of the brain. PLA-04-01 at 19 months old before entering the trial (**A**,**C**) and at 36 months old after treatment with 5-ALA and SFC for 17 months (**B**,**D**). T2-weighted MRI showed high signal intensity in the bilateral basal ganglia (lateral thalamus) and brainstem (substantia nigra) before the trial (**A**,**C**), which decreased after the trial (**B**,**D**). The arrows indicate those legions before and after the trial.

In addition to the clinical improvements suggested by the NPMDS scores, the radiological findings further support the potential efficacy of SPP-004. On the T2-weighted MRI of the PLA-04-01, a high signal intensity was observed in the bilateral basal ganglia (lateral thalamus) and brainstem (substantia nigra) before the clinical trial (Figure 5A,C), whereas these abnormal signals showed marked improvement after the trial (Figure 5B,D), suggesting a therapeutic effect. Such radiological improvement is particularly notable in LS, where the lesions in the basal ganglia are considered hallmark findings and typically persist or worsen over time in the absence of effective treatment. Therefore, the observed changes may provide visual and objective evidence of the therapeutic impact of SPP-004.

Ogawa et al. [28] reported that *MT-ATP6* or *MT-ND5* deficiency induced a severe LS form, whereas the patients with *NDUFAF6*, *ECHS1*, or *SURF1* deficiency had relatively mild symptoms and better survival. However, our trial did not observe a similar association between the causative gene mutations and symptoms, probably due to the limited number of patients. The reduction in the NPMDS scores varied according to the mutation site and onset age. The patients with late-onset disease or nucDNA mutations showed a greater reduction in the NPMDS scores than the patients with early-onset disease or mtDNA mutations. Furthermore, Shimura et al. showed that the effects of 5-ALA and SFC on the patient-derived fibroblasts varied based on the patient’s characteristics [13]. Therefore, the efficacy of SPP-004 may vary depending on the onset age or causative gene mutations.

In this trial, efficacy was only evaluated in children with LS. However, considering that 5-ALA HCl and SFC increased the oxygen consumption rate and ATP production by increasing the MRC complex components in fibroblasts derived from patients with MDs other than LS (including infantile mitochondrial diseases) [13], a similar efficacy could be expected for other pathological types of MDs.

Regarding the surrogate markers, the blood FGF21 level is considered a predictable marker for disease severity [29,30,31,32]. Therefore, we evaluated changes in circulating FGF21 concentration (Figure S3). Extremely high blood FGF21 level in the ALA-02-02 was suggested to be associated with severe symptoms and early death, but in most patients, there was no clear correlation with a change in FGF21 and NPMDS scores. The FGF21 level may not reflect much improvement in symptoms because they are also influenced by confounding factors such as dysfunction of the liver or kidney and concomitant medications.

The present study has several limitations. First, the number of patients was too small to determine a difference in the efficacy between the groups, and patient age was limited to ≤2 years at the time of enrolment. Although we conducted follow-up treatment and care of the enrolled LS patients for 228 weeks, we cannot speculate on the efficacy of SPP-004 for LS patients aged >5 years. The small number of patients led to another limitation of imbalance in severity, with higher baseline NPMDS scores in the SPP-004 group. Second, our study had a short double-blind period of 12 weeks, which made it difficult to observe the long-term efficacy of the study drug in comparison to the placebo. Therefore, the next confirmatory trial was designed to have a longer double-blind period (48 weeks) and a larger number of MD patients, including LS patients with various age groups, i.e., infants, children, and adults. NPMDS section II and section III items 1-2 are unsuitable for short-term evaluation, as mentioned above. Therefore, in the next trial, the total score of NPMDS section I and section III items 3–8 will be employed as the primary endpoint. The results of the next study will be reported elsewhere. The lack of a carcinogenicity study is a safety limitation that must be addressed before application for the approval of SPP-004 as a therapeutic agent.

## 5. Conclusions

The preliminary safety and efficacy of 5-ALA HCl and SFC in pediatric LS patients were demonstrated in our 24-week exploratory initial trial, followed by a long-term study reaching up to 204 weeks. The safety and effectiveness of SPP-004 in patients with MDs, including LS, need to be further investigated in the following trial using the total score of NPMDS section I and section III items 3–8 as the primary endpoint.

## Figures and Tables

**Table 1:** Summary of demographic and other baseline characteristics (SPED-ALA-001 and FAS).

	SPP-004 (*n* = 5)	Placebo (*n* = 5)
Sex—*n* (%)		
Male	3 (60.0)	1 (20.0)
Female	2 (40.0)	4 (80.0)
Age at informed consent—months		
*n*	5	4
Mean ± SD	19.4 ± 2.88	19.5 ± 3.11
Median [Min, Max]	20.0 [15, 23]	19.5 [16, 23]
Presence of preterm birth—*n* (%)		
None	5 (100.0)	4 (80.0)
Yes	0 (0.0)	1 (20.0)
Adjusted age—months		
*n*	0	1
Mean	-	16
Median [Min, Max]	-	16.0 [16, 16]
Primary disease—*n* (%)		
Chronic progressive external ophthalmoplegia	0 (0.0)	0 (0.0)
Myoclonus epilepsy syndrome	0 (0.0)	0 (0.0)
Leigh syndrome	5 (100.0)	5 (100.0)
Leber hereditary optic neuropathy	0 (0.0)	0 (0.0)
Pearson syndrome	0 (0.0)	0 (0.0)
Presence of consanguineous marriage—*n* (%)		
None	5 (100.0)	5 (100.0)
Yes	0 (0.0)	0 (0.0)
Family history—*n* (%)		
None	2 (40.0)	3 (60.0)
Yes	3 (60.0)	2 (40.0)
Medical history—*n* (%)		
None	5 (100.0)	3 (60.0)
Yes	0 (0.0)	2 (40.0)
Presence of complications—*n* (%)		
None	0 (0.0)	2 (40.0)
Yes	5 (100.0)	3 (60.0)

**Table 2:** Baseline characteristics of each patient (SPED-ALA-001).

	SPP-004					Placebo				
Pt No.	ALA-02-01	ALA-02-02 *	ALA-07-01	ALA-09-01	ALA-10-01	PLA-04-01	PLA-04-02	PLA-05-01	PLA-06-01	PLA-08-01
Sex	M	F	F	M	M	F	M	F	F	F
Primary disease	LS	LS	LS	Non-Mito LS	LS	LS	LS	LS	Non-Mito LS	LS
Onset age	0Y 2M	0Y 1M	0Y 11M	0Y 2M	1Y 1M	0Y10M	0Y 5M	0Y 10M	0Y 10M	0Y 1M
Age at start of trial	1Y 7M	1Y 3M	1Y 11M	1Y 9M	1Y 9M	1Y 10M	1Y 10M	2Y 2M	1Y 8M	2Y 0M
MRC activity										n.d.
Sample tissue	Fb	Fb	Fb	Mb	Mb	Fb	Mb	Mb	Fb	
Abnormality	-	I	I	IV	I	I	II	IV	IV	
Gene	*MT-ND6* (T14487C)	*MT-ND3* (T10158C)	*ECHS1*	*SCN8A*	*MT-ND5* (T13094C)	*NDUFV2*	*TRMT5*	*SURF1*	n.d.	*MT-ATP6* (T8993C)
MRI abnormality	Basal ganglia lesion MRS lactate peak	Basal ganglia lesion	Basal ganglia lesion	Basal ganglia lesion	Basal ganglia lesion	Basal ganglia lesion	Basal ganglia lesion	Basal ganglia lesion	Basal ganglia lesion	Basal ganglia lesion
NPMDS I+III3-8	19	25	6	23	7	10	15	16	3	11
Lactate (mmol/L)	2.1 +	3.0 +	2.1 +	1.9	3.2 +	4.1 +	1.5	1.8	1.8	3.0 +
Pyruvate (mmol/L)	0.02	0.09	0.13	0.07	0.15	0.21	0.06	0.09	0.08	0.13
L/P ratio	97.0 +	35.5 +	16.2	27.9 +	21.0 +	19.8	27.8 +	20.3 +	23.3 +	22.7 +
FGF21 (pg/mL)	891.640	10262.091	≤60	96.747	≤60	312.731	569.603	833.855	≤60	≤60

* Died during the double-blind period. F, female; M, male; Fb, fibroblast; Mb, myoblast; n.d., not determined; L/P ratio, ratio of lactate to pyruvate; +, higher than the threshold (lactate > 2.0 mmol/L, L/P ratio > 20).

**Table 3:** Pharmacokinetics of 5-ALA and PPIX in patients.

	*n*	Mean ± SD	Median [Min, Max]
**5-ALA**			
C_max_ (μmol/L)	8	7.00 ± 3.42	6.92 [2.48, 12.3]
t_max_ (h)	8	0.9 ± 0.2	1.0 [0.5, 1.0]
t_1/2_ (h)	-	n.c.	n.c.
AUC_0-8_ (h·μmol/L)	8	12.8 ± 4.14	11.7 [8.13, 18.6]
AUC_0-t_ (h·μmol/L)	8	10.4 ± 3.58	10.3 [6.63, 15.7]
AUC_0-∞_ (h·μmol/L)	-	n.c.	n.c.
**PPIX**			
C_max_ (nmol/L)	8	22.8 ± 16.3	17.0 [6.58, 49.2]
t_max_ (h)	8	3.0 ± 1.1	3.0 [2.0, 4.0]
t_1/2_ (h)	4	1.8 ± 0.3	1.9 [1.4, 2.2]
AUC_0-8_ (h·nmol/L)	8	94.3 ± 64.5	68.3 [31.2, 197]
AUC_0-t_ (h·nmol/L)	8	94.3 ± 64.5	68.3 [31.2, 197]
AUC_0-∞_ (h·nmol/L)	4	76.3 ± 35.7	68.1 [43.5, 125]

n.c.: not calculated.

**Table 4:** Summary of AEs (SP).

**Initial study (SPED-ALA-001)**												
	SPP-004						Placebo					
	Double-blind period (*n*= 5)		Open-label period (*n* = 4)		Total (*n* = 5)		Double-blind period (*n* = 5)		Open-label period (*n* = 5)		Total (*n* = 5)	
	Cases *n* (%)	Events *n*	Cases *n* (%)	Events *n*	Cases *n* (%)	Events *n*	Cases *n* (%)	Events *n*	Cases *n* (%)	Events *n*	Cases *n* (%)	Events *n*
All AEs	5 (100.0)	17	4 (100.0)	12	5 (100.0)	29	5 (100.0)	21	5 (100.0)	24	5 (100.0)	45
Severity												
Mild	2 (40.0)	7	3 (75.0)	9	2 (40.0)	16	2 (40.0)	18	2 (40.0)	17	1 (20.0)	35
Moderate	2 (40.0)	6	1 (25.0)	3	2 (40.0)	9	3 (60.0)	3	3 (60.0)	7	4 (80.0)	10
Severe	1 (20.0)	4	0 (0.0)	0	1 (20.0)	4	0 (0.0)	0	0 (0.0)	0	0 (0.0)	0
Relationship with investigational drug												
Related	2 (40.0)	4	2 (50.0)	2	4 (80.0)	6	1 (20.0)	1	3 (60.0)	3	3 (60.0)	4
Unrelated	3 (60.0)	13	2 (50.0)	10	1 (20.0)	23	4 (80.0)	20	2 (40.0)	21	2 (40.0)	41
Death	1 (20.0)	-	0 (0.0)	-	1 (20.0)	-	0 (0.0)	-	0 (0.0)	-	0 (0.0)	-
Serious AEs	2 (40.0)	7	2 (50.0)	5	3 (60.0)	12	3 (60.0)	3	1 (20.0)	5	3 (60.0)	8
AEs leading to discontinuation	0 (0.0)	0	0 (0.0)	0	0 (0.0)	0	0 (0.0)	0	0 (0.0)	0	0 (0.0)	0
**Long-term administration study (SPED-ALA-002)**												
	All (*n* = 9)											
	Cases—*n* (%)			Events—*n*								
All AEs	9 (100.0)			255								
Severity												
Mild	1 (11.1)			168								
Moderate	3 (33.3)			79								
Severe	5 (55.6)			8								
Relationship with investigational drugs												
Unelated	7 (77.8)			251								
Related	2 (22.2)			4								
Death	1 (11.1)			-								
Serious AEs	7 (77.8)			61								
AEs leading to discontinuation	2 (22.2)			2								

**Table 5:** Incidence of ADRs by SOC and PT (SP).

**Initial Study (SPED-ALA-001)**														
			SPP-004						Placebo					
			Double-blind period		Open-label period		Total		Double-blind period		Open-label period		Total	
			*n* = 5		*n* = 4		*n* = 5		*n* = 5		*n* = 5		*n* = 5	
SOC			Cases	Events	Cases	events	Cases	events	Cases	events	Cases	events	Cases	events
	PT (Med DRA/J Ver 19.0)	Severity	*n* (%)	*n*	*n* (%)	*n*	*n* (%)	*n*	*n* (%)	*n*	*n* (%)	*n*	*n* (%)	*n*
All			2 (40.0)	4	2 (50.0)	2	4 (80.0)	6	1 (20.0)	1	3 (60.0)	3	3 (60.0)	4
Cardiac disorders			1 (20.0)	1	0 (0.0)	0	1 (20.0)	1	0 (0.0)	0	0 (0.0)	0	0 (0.0)	0
	Left ventricular failure	Severe	1 (20.0)	1	0 (0.0)	0	1 (20.0)	1	0 (0.0)	0	0 (0.0)	0	0 (0.0)	0
Gastrointestinal disorder			1 (20.0)	1	1 (25.0)	1	2 (40.0)	2	1 (20.0)	1	1 (20.0)	1	2 (40.0)	2
	Diarrhea	Mild	1 (20.0)	1	0 (0.0)	0	1 (20.0)	1	1 (20.0)	1	0 (0.0)	0	1 (20.0)	1
	Hematochezia	Mild	0 (0.0)	0	1 (25.0)	1	1 (20.0)	1	0 (0.0)	0	0 (0.0)	0	0 (0.0)	0
	Tooth discoloration	Mild	0 (0.0)	0	0 (0.0)	0	0 (0.0)	0	0 (0.0)	0	1 (20.0)	1	1 (20.0)	1
Investigations			0 (0.0)	0	1 (25.0)	1	1 (20.0)	1	0 (0.0)	0	0 (0.0)	0	0 (0.0)	0
	Electroencephalogram abnormal	Mild	0 (0.0)	0	1 (25.0)	1	1 (20.0)	1	0 (0.0)	0	0 (0.0)	0	0 (0.0)	0
Respiratory, thoracic, and mediastinal disorders			1 (20.0)	2	0 (0.0)	0	1 (20.0)	2	0 (0.0)	0	0 (0.0)	0	0 (0.0)	0
	Pulmonary alveolar hemorrhage	Severe	1 (20.0)	1	0 (0.0)	0	1 (20.0)	1	0 (0.0)	0	0 (0.0)	0	0 (0.0)	0
	Respiratory failure	Severe	1 (20.0)	1	0 (0.0)	0	1 (20.0)	1	0 (0.0)	0	0 (0.0)	0	0 (0.0)	0
Skin and subcutaneous tissue disorders			0 (0.0)	0	0 (0.0)	0	0 (0.0)	0	0 (0.0)	0	2 (40.0)	2	2 (40.0)	2
	Urticarias	Moderate	0 (0.0)	0	0 (0.0)	0	0 (0.0)	0	0 (0.0)	0	2 (40.0)	2	2 (40.0)	2
**Long-term administration study (SPED-ALA-002)**														
SOC			Cases	Events										
	PT (MedDRA/J Ver. 22.1)	Severity	*n* (%)	*n*										
All			2 (22.2)	4										
Skin and subcutaneous tissue disorders														
	Urticaria	Moderate	2 (22.2)	2										
		Mild	2 (22.2)	2										

## Data Availability

The data that support this study’s findings are available from the corresponding author upon reasonable request.

## Supplementary Materials

The following supporting information can be downloaded at: https://www.mdpi.com/article/10.3390/life15081168/s1, Figure S1: Changes in total NPMDS scores (section I and section III items 3-8) classified by age of onset (initial study); Figure S2: Changes in total NPMDS scores (section I and section III items 3-8) classified by the locus of mutation (mitochondrial DNA/nuclear DNA); Figure S3: Changes in serum FGF21 of each patient; Table S1: Inclusion criteria and exclusion criteria (SPED-ALA-001 study); Table S2: Incidence of SAEs by SOC and PT (SP and SPED-ALA-001); Table S3: Incidence of SAEs by SOC and PT (SP and SPED-ALA-002); Table S4: Total NPMDS scores (section I and section III items 3-8) during the initial and long-term studies.

## Abbreviations

The following abbreviations are used in this manuscript:

ADR	Adverse drug reaction
AE	Adverse event
5-ALA	5-Aminolevulinic acid
ALT	Alanine aminotransferase
AST	Aspartate aminotransferase
ATR-X	Alpha thalassemia/mental retardation syndrome X-linked
FAS	Full analysis set
FGF21	Fibroblast growth factor 21
FXTAS	Fragile X-associated tremor/ataxia syndrome
GGT	Gamma-glutamyl transferase
LS	Leigh syndrome
MD	Mitochondrial disease
MELAS	Mitochondrial myopathy encephalopathy, lactic acidosis, and stroke-like episodes
MRC	Mitochondrial respiratory chain
MTD	Maximally tolerated dose
Non-Mito LS	Leigh syndrome due to a non-mitochondrial gene variant
NPMDS	Newcastle Pediatric Mitochondrial Disease Scale
SAE	Serious adverse event
SD	Standard deviation
SFC	Sodium ferrous citrate
SOC	System organ class
PPIX	Protoporphyrin IX
PPS	Per protocol set
PT	Preferred term

## Appendix A

### Appendix A.1

**Table A1:** Summary of AEs in Caucasian and Japanese adult subjects after multiple oral doses of 5-ALA/SFC (SPP1C301 study).

	Caucasian				Japanese			
Dose of 5-ALA/SFC		AEs		SAEs		AEs		SAEs
	Cases	% (Cases)	Events		Cases	% (Cases)	Events	
Placebo	(*n* = 6)	50.0% (3/6)	5	0	(*n* = 4)	50.0% (2/4)	2	0
150 mg/235 mg	(*n* = 6)	50.0% (3/6)	4	0	(*n* = 6)	50.0% (3/6)	3	0
300 mg/470 mg	(*n* = 6)	66.7% (4/6)	11	0	(*n* = 6)	0.0% (0/6)	0	0
600 mg/940 mg	(*n* = 6)	83.3% (5/6)	19	0				

N = number of subjects

### Appendix A.2

**Table A2:** Summary of ADRs by name in Caucasian and Japanese adult subjects after multiple oral doses of 5-ALA/SFC (SPP1C301 study).

	Caucasian				Japanese		
	Placebo	150 mg/235 mg	300 mg /470 mg	600 mg /940 mg	Placebo	150 mg /235 mg	300 mg /470 mg
	(*n* = 6)	(*n* = 6)	(*n* = 6)	(*n* = 6)	(*n* = 6)	(*n* = 6)	(*n* = 6)
Abdominal distension			2			2	
Nausea		1	2	1			
Feces discolored			2	1			
Headache	1	1		2			
Dizziness			1	1			
Chest discomfort				2			
Myalgia		1		1			

### Appendix A.3

**Table A3:** Dosage and administration of investigational drug.

Body Weight	Daily Dose (5-ALA/SFC)	Number of Doses and Number of Capsules Taken for 5-ALA and SFC
>20 kg	50 mg/78.44 mg	Twice a day, 1 capsule in the morning and 1 capsule in the evening
≥20 kg and <30 kg	75 mg/117.66 mg	Twice a day, 2 capsules in the morning and 1 capsule in the evening
≥30 kg and <40 kg	100 mg/156.88 mg	Twice a day, 2 capsules in the morning and 2 capsules in the evening
≥40 kg and <50 kg	125 mg/196.1 mg	Twice a day, 3 capsules in the morning and 2 capsules in the evening
≥50 kg	150 mg/235.32 mg	Twice a day, 3 capsules in the morning and 3 capsules in the evening

## References

[1] Russell O.M., Gorman G.S., Lightowlers R.N., Turnbull D.M. (2020). Mitochondrial Diseases: Hope for the Future. Cell.

[2] Thompson K., Collier J.J., Glasgow R.I.C., Robertson F.M., Pyle A., Blakely E.L., Alston C.L., Oláhová M., McFarland R., Taylor R.W. (2020). Recent advances in understanding the molecular genetic basis of mitochondrial disease. J. Inherit. Metab. Dis..

[3] Rodenburg R.J. (2011). Biochemical diagnosis of mitochondrial disorders. J. Inherit. Metab. Dis..

[4] Saneto R.P., Friedman S.D., Shaw D.W. (2008). Neuroimaging of mitochondrial disease. Mitochondrion.

[5] Schubert Baldo M., Vilarinho L. (2020). Molecular basis of Leigh syndrome: A current look. Orphanet J. Rare Dis..

[6] Diodato D., Schiff M., Cohen B.H., Bertini E., Rahman S., Workshop participants (2023). 258th ENMC international workshop Leigh syndrome spectrum: Genetic causes, natural history and preparing for clinical trials 25–27 March 2022, Hoofddorp, Amsterdam, The Netherlands. Neuromuscul. Disord..

[7] Barcelos I., Shadiack E., Ganetzky R.D., Falk M.J. (2020). Mitochondrial medicine therapies: Rationale, evidence, and dosing guidelines. Curr. Opin. Pediatr..

[8] Almannai M., El-Hattab A.W., Ali M., Soler-Alfonso C., Scaglia F. (2020). Clinical trials in mitochondrial disorders, an update. Mol. Genet. Metab..

[9] Parikh S., Goldstein A., Koenig M.K., Scaglia F., Enns G.M., Saneto R., Anselm I., Cohen B.H., Falk M.J., Greene C. (2015). Diagnosis and management of mitochondrial disease: A consensus statement from the Mitochondrial Medicine Society. Genet. Med..

[10] Koga Y., Povalko N., Inoue E., Nakamura H., Ishii A., Suzuki Y., Yoneda M., Kanda F., Kubota M., Okada H. (2018). Therapeutic regimen of L-arginine for MELAS: 9-year, prospective, multicenter, clinical research. J. Neurol..

[11] Ikawa M., Povalko N., Koga Y. (2020). Arginine therapy in mitochondrial myopathy, encephalopathy, lactic acidosis, and stroke-like episodes. Curr. Opin. Clin. Nutr. Metab. Care.

[12] Nishio Y., Fujino M., Zhao M., Ishii T., Ishizuka M., Ito H., Takahashi K., Abe F., Nakajima M., Tanaka T. (2014). 5-Aminolevulinic acid combined with ferrous iron enhances the expression of heme oxygenase-1. Int. Immunopharmacol..

[13] Shimura M., Nozawa N., Ogawa-Tominaga M., Fushimi T., Tajika M., Ichimoto K., Matsunaga A., Tsuruoka T., Kishita Y., Ishii T. (2019). Effects of 5-aminolevulinic acid and sodium ferrous citrate on fibroblasts from individuals with mitochondrial diseases. Sci. Rep..

[14] Ogura S.-I., Maruyama K., Hagiya Y., Sugiyama Y., Tsuchiya K., Takahashi K., Abe F., Tabata K., Okura I., Nakajima M. (2011). The effect of 5-aminolevulinic acid on cytochrome c oxidase activity in mouse liver. BMC Res. Notes.

[15] Shioda N., Yabuki Y., Yamaguchi K., Onozato M., Li Y., Kurosawa K., Tanabe H., Okamoto N., Era T., Sugiyama H. (2018). Targeting G-quadruplex DNA as cognitive function therapy for ATR-X syndrome. Nat. Med..

[16] Asamitsu S., Yabuki Y., Ikenoshita S., Kawakubo K., Kawasaki M., Usuki S., Nakayama Y., Adachi K., Kugoh H., Ishii K. (2021). CGG repeat RNA G-quadruplexes interact with FMRpolyG to cause neuronal dysfunction in fragile X-related tremor/ataxia syndrome. Sci. Adv..

[17] Suzuki H., Masuki S., Morikawa A., Ogawa Y., Kamijo Y.I., Takahashi K., Nakajima M., Nose H. (2018). Effects of 5-aminolevulinic acid supplementation on home-based walking training achievement in middle-aged depressive women: Randomized, double-blind, crossover pilot study. Sci. Rep..

[18] Perez M.H., Shintani T.T., Rodriguez B.L., Davis J., Harrigan R.C. (2013). The Role of 5-aminolevulinic acid (5-ALA) and sleep. Int. J. Clin. Med..

[19] Aquino R.K., Perez M., Sil P., Shintani T., Harrigan R., Rodriguez B. (2018). The Relationship of 5-Aminolevulinic Acid on Mood and Coping Ability in Prediabetic Middle Aged and Older Adults. Geriatrics.

[20] Kohda M., Tokuzawa Y., Kishita Y., Nyuzuki H., Moriyama Y., Mizuno Y., Hirata T., Yatsuka Y., Yamashita-Sugahara Y., Nakachi Y. (2016). A Comprehensive Genomic Analysis Reveals the Genetic Landscape of Mitochondrial Respiratory Chain Complex Deficiencies. PLoS Genet..

[21] Mingone C.J., Gupte S.A., Chow J.L., Ahmad M., Abraham N.G., Wolin M.S. (2006). Protoporphyrin IX generation from delta-aminolevulinic acid elicits pulmonary artery relaxation and soluble guanylate cyclase activation. Am. J. Physiol. Lung Cell. Mol. Physiol..

[22] Juzenas P., Juzeniene A. (2010). Reduction of cutaneous photosensitivity by application of ointment containing ferrous or cobaltous ions concomitant with the use of topical protoporphyrin IX precursors. Photodiagnosis Photodyn. Ther..

[23] Hayashi M., Fukuhara H., Inoue K., Shuin T., Hagiya Y., Nakajima M., Tanaka T., Ogura S.-I., Missirlis F. (2015). The effect of iron ion on the specificity of photodynamic therapy with 5-aminolevulinic acid. PLoS ONE.

[24] Ota U., Fukuhara H., Ishizuka M., Abe F., Kawada C., Tamura K., Tanaka T., Inoue K., Ogura S.-I., Shuin T. (2015). Plasma protoporphyrin IX following administration of 5-aminolevulinic acid as a potential tumor marker. Mol. Clin. Oncol..

[25] Phoenix C., Schaefer A., Elson J., Morava E., Bugiani M., Uziel G., Smeitink J., Turnbull D., McFarland R. (2006). A scale to monitor progression and treatment of mitochondrial disease in children. Neuromuscul. Disord..

[26] Baertling F., Rodenburg R.J., Schaper J., Smeitink J.A., Koopman W.J.H., Mayatepek E., Morava E., Distelmaier F. (2014). A guide to diagnosis and treatment of Leigh syndrome. J. Neurol. Neurosurg. Psychiatry.

[27] Lim A.Z., Ng Y.S., Blain A., Jiminez-Moreno C., Alston C.L., Nesbitt V., Simmons L., Santra S., Wassmer E., Blakely E.L. (2022). Natural History of Leigh Syndrome: A Study of Disease Burden and Progression. Ann. Neurol..

[28] Ogawa E., Fushimi T., Ogawa-Tominaga M., Shimura M., Tajika M., Ichimoto K., Matsunaga A., Tsuruoka T., Ishige M., Fuchigami T. (2020). Mortality of Japanese patients with Leigh syndrome: Effects of age at onset and genetic diagnosis. J. Inherit. Metab. Dis..

[29] Suomalainen A., Elo J.M., Pietiläinen K.H., Hakonen A.H., Sevastianova K., Korpela M., Isohanni P., Marjavaara S.K., Tyni T., Kiuru-Enari S. (2011). FGF-21 as a biomarker for muscle-manifesting mitochondrial respiratory chain deficiencies: A diagnostic study. Lancet Neurol..

[30] Su S.-L., Wang W.-F., Wu S.-L., Wu H.-M., Chang J.-C., Huang C.-S., Cheng W.-L., Soong B.-W., Lee Y.-C., Li J.-Y. (2012). FGF21 in ataxia patients with spinocerebellar atrophy and mitochondrial disease. Clin. Chim. Acta.

[31] Lehtonen J.M., Forsström S., Bottani E., Viscomi C., Baris O.R., Isoniemi H., Höckerstedt K., Österlund P., Hurme M., Jylhävä J. (2016). FGF21 is a biomarker for mitochondrial translation and mtDNA maintenance disorders. Neurology.

[32] Li Y., Li S., Qiu Y., Zhou M., Chen M., Hu Y., Hong S., Jiang L., Guo Y. (2022). Circulating FGF21 and GDF15 as Biomarkers for Screening, Diagnosis, and Severity Assessment of Primary Mitochondrial Disorders in Children. Front. Pediatr..

