# Supplementary material for: Efficacy and Safety of 5-Aminolevulinic Acid Hydrochloride Combined with Sodium Ferrous Citrate in Pediatric Patients with Leigh Syndrome and Central Nervous System Disorders: An Initial Exploratory Trial with a Double-Blind Placebo-Controlled Period, Followed by an Open-Label Period and a Subsequent Long-Term Administration Study
Source: Life (Basel). 2025 Jul 23;15(8):1168. doi: 10.3390/life15081168 (PMC12387954; doi:10.3390/life15081168)
Supplement: Supplementary file 1 [file life-15-01168-s001.zip › life-3736708-supplementary.pdf]

Supplemental material

Efficacy and safety of 5-aminolevulinic acid hydrochloride combined with sodium ferrous citrate on pediatric patients with Leigh syndrome who developed central nervous system disorders: An initial exploratory trial with a double-blind placebo-controlled period, followed by an open-label period and a subsequent long-term administration study.

Figure S1. Changes in total NPMDS scores (section I and section III items 3-8) classified by age of onset (Initial study) . 2

Figure S2. Changes in total NPMDS scores (section I and section III items 3-8) classified by locus of mutation (mitochondrial DNA/nuclear DNA). 3

Figure S3. Changes in serum FGF21 of each patient. 5

Table S1. Inclusion criteria and exclusion criteria (SPED-ALA-001 study). 6

Table S2. Incidence of SAEs by SOC and PT (SP, SPED-ALA-001). 7

Table S3. Incidence of SAEs by SOC and PT (SP, SPED-ALA-002) 8

Table S4. NPMDS total score section I and section III items 3-8 during the initial and long-term studies. 9

Figure S1.

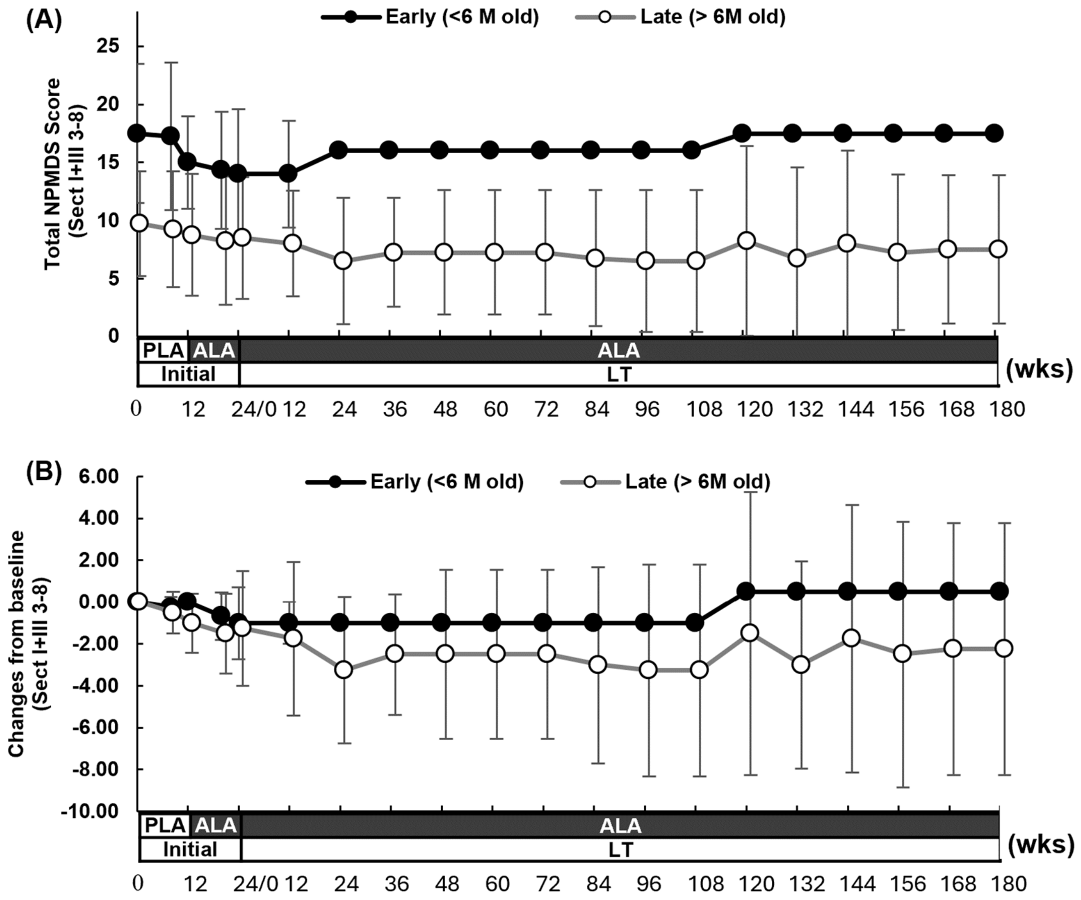

Figure S1. Changes in total NPMDS scores (section I and section III items 3-8) classified by age of onset (Initial study).

(A) Changes in total NPMDS scores (section I and section III items 3-8). (B). Changes from baseline scores. Average± Standard deviation of early-onset group (< 6-month, filled-circle) and late-onset group (> 6-month, Open-circle) are shown. Error bars were not shown if the number of patients was 3 or less.

Figure S2.

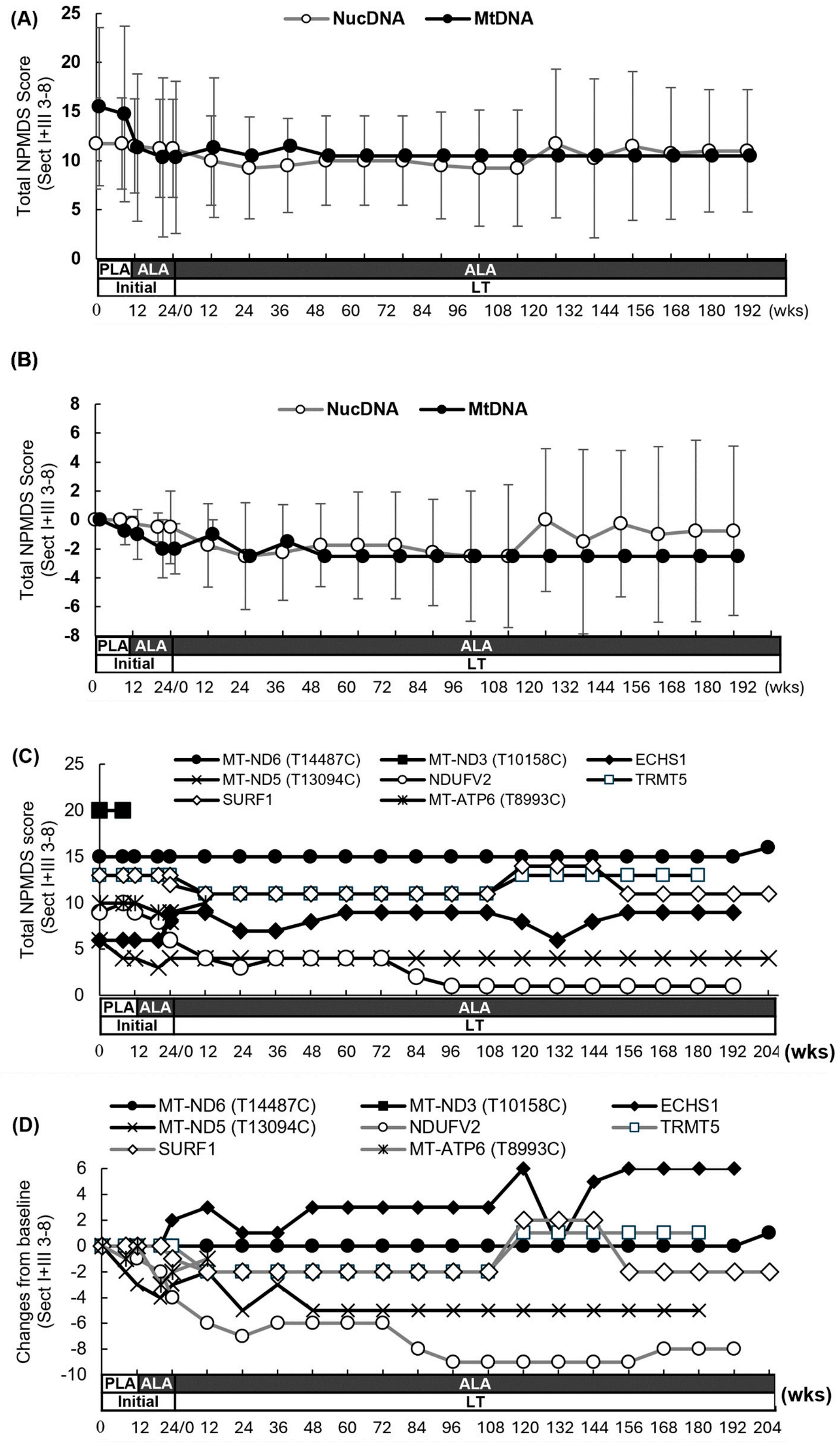
(A)
NucDNA
MtDNA
Total NPMDS Score (Sect I+III 3-8)
25
20
15
10
5
0
PLA
ALA
Initial
LT
0 12 24/0 12 24 36 48 60 72 84 96 108 120 132 144 156 168 180 192 (wks)
(B)
NucDNA
MtDNA
Total NPMDS Score (Sect I+III 3-8)
8
6
4
2
0
-2
-4
-6
-8
PLA
ALA
Initial
LT
0 12 24/0 12 24 36 48 60 72 84 96 108 120 132 144 156 168 180 192 (wks)
(C)
MT-ND6 (T14487C)
MT-ND3 (T10158C)
ECHS1
MT-ND5 (T13094C)
NDUFV2
TRMT5
SURF1
MT-ATP6 (T8993C)
Total NPMDS score (Sect I+III 3-8)
25
20
15
10
5
0
PLA
ALA
Initial
LT
0 12 24/0 12 24 36 48 60 72 84 96 108 120 132 144 156 168 180 192 204 (wks)
(D)
MT-ND6 (T14487C)
MT-ND3 (T10158C)
ECHS1
MT-ND5 (T13094C)
NDUFV2
TRMT5
SURF1
MT-ATP6 (T8993C)
Changes from baseline (Sect I+III 3-8)
6
4
2
0
-2
-4
-6
-8
-10
PLA
ALA
Initial
LT
(wks)
0 12 24/0 12 24 36 48 60 72 84 96 108 120 132 144 156 168 180 192 204

Figure S2. Changes in total NPMDS scores (section I and section III items 3-8) classified by locus of mutation (mitochondrial DNA/nuclear DNA).

(A and C) Changes in total NPMDS scores (section I and section III items 3-8). (B and D). Changes from baseline scores. average± Standard deviation (A and B) and change in score of each patient (C and D) and of nuclear DNA mutation group (solid line) and mitochondrial DNA mutation group (dotted line) are shown. Nuclear mutations: *ECS1*, *NDUFV2*, *TRMT5*, *SURF1*; mitochondrial mutations: *MT-ND6*, *MT-ND3, MT-ND5*, *MT-ATP6*; nucDNA: nuclear DNA mutation, mtDNA: mitochondrial DNA mutation.

Figure S3.

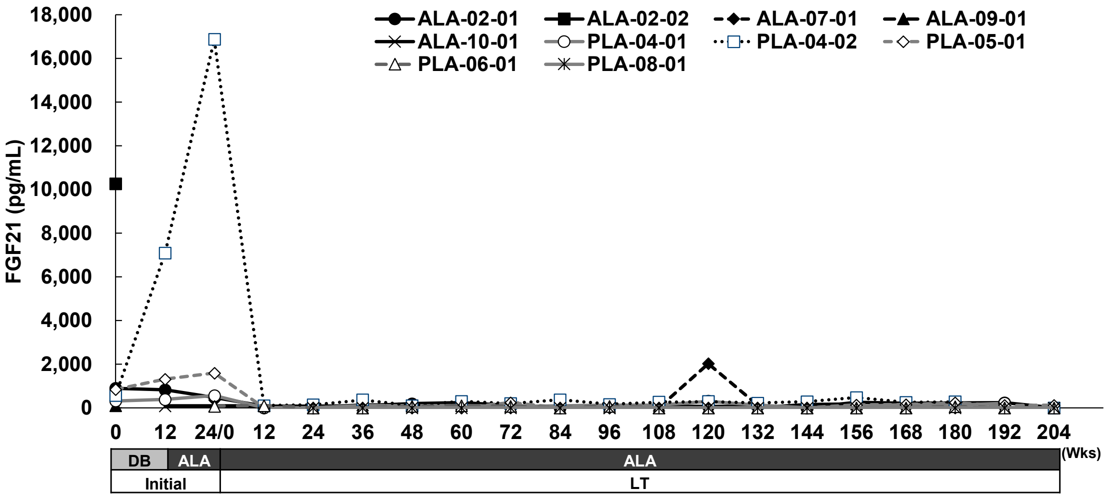

Figure S3. Changes in serum FGF21 of each patient. DB: double-blind period, ALA: SPP-004, Initial: SPED-ALA-001 study, LT: SPED-ALA-002 study.

Table S1. Inclusion criteria and exclusion criteria (SPED-ALA-001 study).

Inclusion criteria

Japanese patients who meet all the following criteria

1) Patients who were confirmed to be respiratory chain enzyme deficiency or mitochondrial gene abnormality by following clinical findings in patients with suspected mitochondrial disease*

   *Patients with all the following 1 to 3, as well as one or more of the conditions in 4

   1. Progressive neurological disorders associated with delayed motor and mental development
   2. Symptoms or signs of disease in brain stem and/or cerebral basal ganglia
   3. Increased lactate levels in blood and/or cerebrospinal fluid
   4. Patients who meet one or more of the followings:

      I. Patients who have characteristic findings in images. (bilateral and symmetrical lesion of the brain stem or basal ganglia)

      II. Patients with typical neuropathological changes (spongiform degeneration)

      III. Patients with a sibling with similar symptoms

2) Patients aged 3 months and < 2 years at the acquisition of consent forms (premature infants are dealt as corrected age in month)

3) Patients with blood lactate-pyruvate ratio ?15.0

4) Patients with written informed consent from their legal representatives (persons in parental authority or guardians) *

Exclusion criteria

Patients who meet any of the following criteria are excluded from this trial.

1) Patients with mitochondrial cardiomyopathy

2) Patients with severe cardiac function or renal function disorders

3) Patients complicated by sepsis

4) Patients with a history of drug allergy

5) Patients with a history of hypersensitivity to any ingredients of the investigational drugs

6) Patients participated in another clinical study within 12 weeks prior to the informed consent.

7) Patients whom the investigator and sub-investigator considered inappropriate to participating this trial

Table S2. Incidence of SAEs by SOC and PT (SP, SPED-ALA-001).

SOC		SPP-004 group						placebo control group					
		double-blind period N=5		Open-label period N =4		total N =5		double-blind period N =5		Open-label period N =5		total N =5	
		cases	events	cases	events	cases	events	cases	events	cases	events	cases	events
	PT	n (%)	n	n (%)	n	n (%)	n	n (%)	n	n (%)	n	n (%)	n
All		2 (40.0)	7	2 (50.0)	5	3 (60.0)	12	3 (60.0)	3	1 (20.0)	5	3 (60.0)	8
Cardiac disorders		1 (20.0)	1	0 (0.0)	0	1 (20.0)	1	0 (0.0)	0	0 (0.0)	0	0 (0.0)	0
	Left ventricular failure	1 (20.0)	1	0 (0.0)	0	1 (20.0)	1	0 (0.0)	0	0 (0.0)	0	0 (0.0)	0
Infections and infestations		1 (20.0)	2	2 (50.0)	3	2 (40.0)	5	1 (20.0)	1	1 (20.0)	3	2 (40.0)	4
	Bronchitis	1 (20.0)	2	0 (0.0)	0	1 (20.0)	2	1 (20.0)	1	0 (0.0)	0	1 (20.0)	1
	Gastroenteritis	0 (0.0)	0	1 (25.0)	1	1 (20.0)	1	0 (0.0)	0	0 (0.0)	0	0 (0.0)	0
	Gastroenteritis rotavirus	0 (0.0)	0	0 (0.0)	0	0 (0.0)	0	0 (0.0)	0	1 (20.0)	1	1 (20.0)	1
	Nasopharyngitis	0 (0.0)	0	0 (0.0)	0	0 (0.0)	0	0 (0.0)	0	1 (20.0)	1	1 (20.0)	1
	Pharyngitis	0 (0.0)	0	1 (25.0)	1	1 (20.0)	1	0 (0.0)	0	0 (0.0)	0	0 (0.0)	0
	Pneumonia	0 (0.0)	0	1 (25.0)	1	1 (20.0)	1	0 (0.0)	0	1 (20.0)	1	1 (20.0)	1
Metabolic and nutrition disorders		1 (20.0)	1	0 (0.0)	0	1 (20.0)	1	0 (0.0)	0	0 (0.0)	0	0 (0.0)	0
	Hyperglycemia	1 (20.0)	1	0 (0.0)	0	1 (20.0)	1	0 (0.0)	0	0 (0.0)	0	0 (0.0)	0
Nervous system disorders		0 (0.0)	0	0 (0.0)	0	0 (0.0)	0	2 (40.0)	2	1 (20.0)	1	2 (40.0)	3
	Epilepsy	0 (0.0)	0	0 (0.0)	0	0 (0.0)	0	1 (20.0)	1	1 (20.0)	1	1 (20.0)	2
	Generalized tonic-clonic seizure	0 (0.0)	0	0 (0.0)	0	0 (0.0)	0	1 (20.0)	1	0 (0.0)	0	1 (20.0)	1
Respiratory, thoracic and mediastinal disorders		1 (20.0)	3	1 (25.0)	2	2 (40.0)	5	0 (0.0)	0	0 (0.0)	0	0 (0.0)	0
	Asthma	0 (0.0)	0	1 (25.0)	2	1 (20.0)	2	0 (0.0)	0	0 (0.0)	0	0 (0.0)	0
	Hypoxia	1 (20.0)	1	0 (0.0)	0	1 (20.0)	1	0 (0.0)	0	0 (0.0)	0	0 (0.0)	0
	Pulmonary alveolar haemorrhage	1 (20.0)	1	0 (0.0)	0	1 (20.0)	1	0 (0.0)	0	0 (0.0)	0	0 (0.0)	0
	Respiratory failure	1 (20.0)	1	0 (0.0)	0	1 (20.0)	1	0 (0.0)	0	0 (0.0)	0	0 (0.0)	0
Skin and subcutaneous tissue disorders		0 (0.0)	0	0 (0.0)	0	0 (0.0)	0	0 (0.0)	0	1 (20.0)	1	1 (20.0)	1
	Urticaria	0 (0.0)	0	0 (0.0)	0	0 (0.0)	0	0 (0.0)	0	1 (20.0)	1	1 (20.0)	1

SAE, Serious Adverse Event, SOC: System Organ Class, PT: Preferred Term, SP: Safety Population.

Med DRA/J Ver 19.0

Table S3. Incidence of SAEs by SOC and PT (SP, SPED-ALA-002).

SOC	N=9	
P.T.	Cases n (%)	Events n
All	7 (77.8)	61
Infections and infestations	6 (66.7)	27
Pneumonia	3 (33.3)	6
Gastroenteritis	3 (33.3)	5
Bronchitis	2 (22.2)	4
Influenza	2 (22.2)	3
Bronchiolitis	1 (11.1)	1
Clostridium difficile colitis	1 (11.1)	1
Croup infectious	1 (11.1)	1
Epstein-Barr virus infection	1 (11.1)	1
Tracheitis	1 (11.1)	1
Viral infection	1 (11.1)	1
Viral upper respiratory tract infection	1 (11.1)	1
Pneumonia bacterial	1 (11.1)	1
Respiratory syncytial virus bronchitis	1 (11.1)	1
Metabolic and nutritional disorders	1 (11.1)	1
dehydration	1 (11.1)	1
nervous system disorders	2 (22.2)	4
epilepsy	2 (22.2)	4
Respiratory, thoracic and mediastinal disorders	4 (44.4)	20
Pneumonia aspiration	2 (22.2)	5
Upper respiratory tract inflammation	2 (22.2)	5
Asphyxia	1 (11.1)	3
Acute respiratory failure	1 (11.1)	2
Asthma	1 (11.1)	1
Chronic respiratory failure	1 (11.1)	1
Epistaxis	1 (11.1)	1
Hypoxia	1 (11.1)	1
Respiratory failure	1 (11.1)	1
Gastrointestinal disorders	3 (33.3)	6
Vomiting	3 (33.3)	3
Dyspepsia	1 (11.1)	3
General disorders and administration site conditions	1 (11.1)	1
Pyrexia	1 (11.1)	1
Surgical and medical procedures	2 (22.2)	2
Gastrostomy	2 (22.2)	2

SAE: Serious Adverse Event, SOC: System Organ Class, PT: Preferred Term, SP: Safety Population.

MedDRA/J Ver. 22.1

Table S4. NPMDS total score section I and section III items 3-8 during the initial and long-term studies.

		SPP-004 Mean±SD (N)	Placebo Mean±SD (N)	All Mean±SD (N)
Initial study	0 wks	16.0±8.94 (N=5)	11.0±5.15 (N=5)	13.5±7.37 (N=10)
(SPED-ALA-001)	8 wks	15.6±9.48 (N=5)	11.0±4.80 (N=5)	13.3±7.48 (N=10)
	12 wks	12.8±9.07 (N=4)	10.8±5.22 (N=5)	11.7±6.75 (N=9)
	20 wks	12.5±9.40 (N=4)	11.0±4.18 (N=5)	11.7±6.52 (N=9)
	24 wks	13.3±8.62 (N=4)	10.4±4.72 (N=5)	11.7±6.42 (N=9)
Long-term study	0 wks	13.5±8.43 (N=4)	10.4±4.34 (N=5)	11.8±6.22 (N=9)
(SPED-ALA-002)	12 wks	13.5±7.72 (N=4)	9.2±4.55 (N=5)	11.1±6.15 (N=9)
	24 wks	12.3±9.22 (N=4)	8.8±5.56 (N=4)	10.5±7.29 (N=8)
	36 wks	13.0±8.83 (N=4)	9.0±5.23 (N=4)	11.0±7.05 (N=8)
	48 wks	13.0±9.20 (N=4)	10.0±4.55 (N=4)	11.5±6.91 (N=8)
	60 wks	13.3±9.54 (N=4)	10.5±4.51 (N=4)	11.9±7.06 (N=8)
	72 wks	13.3±9.54 (N=4)	11.5±5.07 (N=4)	12.4±7.13 (N=8)
	84 wks	13.3±9.54 (N=4)	11.3±6.29 (N=4)	12.3±7.55 (N=8)
	96 wks	13.3±9.54 (N=4)	11.0±6.78 (N=4)	12.1±7.75 (N=8)
	108 wks	13.3±9.54 (N=4)	9.8±5.97 (N=4)	11.5±7.60 (N=8)
	120 wks	14.0±9.20 (N=4)	11.0±7.70 (N=4)	12.5±8.02 (N=8)
	132 wks	12.5±10.08 (N=4)	11.0±7.70 (N=4)	11.8±8.35 (N=8)
	144 wks	10.7±8.50 (N=3)	11.0±7.70 (N=4)	10.9±7.34 (N=7)
	156 wks	11.0±8.54 (N=3)	10.0±6.68 (N=4)	10.4±6.85 (N=7)
	168 wks	11.0±8.54 (N=3)	10.5±6.19 (N=4)	10.7±6.60 (N=7)
	180 wks	11.0±8.54 (N=3)	10.5±6.19 (N=4)	10.7±6.60 (N=7)
	192 wks	15.5±4.95 (N=2)	8.7±6.11 (N=3)	11.4±6.23 (N=5)
	204 wks	20.0±0.00 (N=1)	12.0±2.83 (N=2)	14.7±5.03 (N=3)
